# Connectivity in support of student co-design of innovative mathematics curriculum trajectories

**DOI:** 10.1007/s11858-021-01297-4

**Published:** 2021-08-27

**Authors:** Birgit Pepin

**Affiliations:** Eindhoven School of Education, Eindhoven University of Technology, Eindhoven, The Netherlands

**Keywords:** Connectivity, Student (co-)design of their own curriculum, Digital curriculum resources, Connected curriculum trajectory

## Abstract

The argument of this theoretical paper is that the existence and availability of suitable digital curriculum resources, accelerated by the recent pandemic, have required a revision of the pedagogical landscape in terms of ways in which students can be empowered to (co-)design their own curriculum trajectories. For this purpose, I argue, students need to be supported in considering many connections, to arrive at coherent trajectories. Based on complexity thinking and curriculum design with digital resources, I propose the concept of connectivity as a crucial principle for creating coherent curriculum trajectories. If students are to become the co-designers of their own curriculum, they need a frame that raises their awareness about the many connections to be made and that supports their capability for actually realizing them. Drawing strongly on my own work and related work by others, I analyse and illustrate the connections made by students, teachers and curriculum designers in their design of mathematics tasks, lessons and learning trajectories with digital resources. Results show that connections can be made at several levels, namely, at a social level, at a material level, at programme level, and at a didactical level. Leaning on systems thinking, connections can be systematically considered, which is likely to help students to enhance the coherence of their designs. I contend that a student-designed ‘connected curriculum trajectory’ is likely to become the focus of future research activities in innovative learning environments: this endeavor would connect aspects of curriculum, mathematical content, learning strategies of students, and the use of new technologies.

## Introduction

While I am writing this article, the whole world is still struggling with the COVID-19 pandemic, which is also changing the perceptions of policy makers and teachers concerning how education should be designed and provided. Moreover, it has changed students’ perceptions of how to learn and study, especially which resources to use beneficially for their learning (e.g., (e-)textbooks, video clips, peers). Certainly, since COVID-19, education has been expected to be provided mainly ‘at a distance’ (via the internet), and technology will play a major role in how education will be provided in the future.

During the pandemic, students had to adjust their learning practices: from being told by the teacher what and how to learn, and how to use the available resources at each particular stage of their learning, to developing more self-directed learning patterns where the teacher takes on the role of tutor through the ‘labyrinth’ of tasks and curriculum resources, and students have to ask for help and advice on how to resolve their questions (Millar et al., 2021). This situation has become even more pertinent at university, where students are expected, and ‘forced’ through the pandemic, to develop their own practices: these include sequencing and aligning different available resources in order to study the mathematics (Biwer et al., 2021; Tyaningsih et al., 2021).

In addition, many recent educational innovations (in schools and universities) aim to foster active, self-regulated and collaborative ways of studying and learning, as these are seen as most beneficial in terms of both the quality of learning (as evidenced in, e.g., student learning outcomes and student learning experiences) and the preparation for life-long learning (Vermunt et al., 2017). Examples of education models with this aim include problem-based learning, project-centered learning, and challenge-based learning, to name but a few.

In traditional lecture–tutor group university mathematics courses, students organize, sequence and align their work with resources in ways that depend, among others, on the course organization and what lecturers asks them to do (Kock & Pepin, 2018). However, in universities of technology in which it has been decided to move towards more Challenge-Based (CB) education, interdisciplinary groups of students work on authentic engineering tasks (Kohn Rådberg et al., 2020). In this type of education students have to become self-regulated, and they have to be able to identify and select the resources necessary for the problem at hand, in order to work on the challenge. Thus far, it is not clear what kinds of frames of support would benefit students in such learning environments.

Increasing students’ self-regulation assumes a gradual decrease in teacher regulation of student learning. This is often contradictory, in particular, to university teachers’ common practices and beliefs concerning how they perceive they have to support their students (Beaudoin, 2009). This means that educational innovations, such as CB education, not only have implications for student learning, and the opportunities they are given to learn, but also for teachers’ teaching practices and the support they may need, to turn from ‘instructor’ to ‘coach’. In more traditional forms of teaching, teachers often regard themselves as discipline specialists, whose main responsibility is to transfer subject knowledge to their students (Anastasakis et al., 2017). Instead, in many innovative learning environments, the teacher’s role gradually changes: from subject matter expert who explains and clarifies the subject matter, to a coach, who is expected to support and guide the active, collaborative and self-regulated learning processes of students (Vermunt et al., 2017). In CB education, for example, teachers are expected to supervise project groups and to facilitate collaborative learning.

Moreover, enhancing student self-regulation in innovative learning environments involves the identification and selection of suitable resources for learning, in other words that students get more active in this task (besides taking on what teachers usually already do). To name but a few, these resources could include the following: resources for distance learning (e.g., e-textbooks); resources for talking to peers and friends (e.g., platforms); resources for learning and applying particular concepts needed to solve the challenge or problem at hand (e.g., videos). Again, support is needed, for example, in how to select suitable resources and for which purpose.

These changes and developments call for different student (and teacher) competences, and these are related to the (re-)design of the curriculum with digital resources. With the change of the nature of textbooks and curriculum resources, new ways of supporting students (and teachers) are needed (Pepin et al., 2016).

To summarize: in order that students actually have the opportunity to develop their own curriculum trajectories, support is needed, also in the form of frameworks for student curriculum (co-)design. Meanwhile, teachers need to be professionally supported (e.g., with such frames), to be able to support students in their endeavors to develop their own active, self-regulated and collaborative ways of studying and learning, and also to coach students through an innovative curriculum. In addition, frameworks that support the analysis of curriculum resources are needed, to see which resources are likely to be beneficial for student curriculum trajectories.

In view of these challenges, the concept of ‘connectivity’ is relevant, although it has been used in many different ways. Drawing strongly on my own work (previously conducted studies) and related work by others, the aim of this theoretical paper is to explore and to give meaning to this concept in innovative learning environments, in terms of how it can support students (and teachers) to become ‘(co-)designers’ of their own curriculum, and how digital curriculum resources can support that process.

The research question is the following:

In which ways can *connectivity* (as a principle) support students to (co-) design their curriculum trajectories in innovative learning environments, and how can it support student interactions with resources in order to create curricular coherence?

After this introductory section, in the second section, I outline the following theoretical frames that I have used: connectivity; resources and their use; and curriculum design. In a brief third section, I outline how I selected three studies for my investigation, and in the fourth section I explore these studies with respect to connectivity, and present the results from these studies. In the fifth section, I answer the research question and present my conclusions.

## Theoretical frames

In this section I explain and define the notions of (1) connectivity; (2) resources and their use; and (3) curriculum design.

(1) *Connectivity*

Connectivity can be regarded as applying at different levels of the teaching and learning enterprise. My ideas on connectivity were inspired by articulations of complexity thinking at theoretical level (e.g., Capra, 1996), from mathematics education research (e.g., Davis & Simmt, 2003; Hiebert & Carpenter, 1992), and from curriculum studies (e.g., Doll, 2008; St. Julien, 2005; Van den Akker, 2003; Van den Akker & Nieveen, 2017).

At the **theoretical level**, connectivity is closely related to systems/complexity thinking (e.g., Capra, 1996), where connections are made in order to develop a *dynamic balance* between system constituents at a certain moment. However, at subsequent moments this balance can be broken due to an increase of energy for one or selected parts of the system. At this moment the *tipping point* is reached and the system ‘crashes’ and attempts to develop a new dynamic balance.

In terms of learning, this system can be interpreted as the process of learning, when new connections are made (i.e., new knowledge is presented to or experienced by the student/s), as ‘old’ connections no longer work, and a new dynamic balance between the ‘previous’ and ‘new’ connections has to be established by the student. Another connected notion within systems thinking is that ‘the whole (system) is more than the sum of its individual parts’. Systems thinking has been illustrated in physics (e.g., in thermodynamics), in school organization/leadership reform (e.g., Fullan, 2007; Morrison, 2002), and also in mathematics education research (e.g., Davis & Simmt, 2003).

Connectivity can also be related to an education theory, the so-called *connectivism* (Dowens, 2007):

At its heart, connectivism is the thesis that knowledge is distributed across a network of connections, and therefore that learning consists of the ability to construct and traverse those networks. It shares with some other theories a core proposition, that knowledge is not acquired, as though it were a thing. (online).

In terms of **mathematics learning**, two aspects of complex systems from the complexity thinking literature are particularly interesting for this paper (e.g., Koch et al., 2018). First, a complex system is a network of interconnected elements or nodes arranged in multiple, co-implicated layers (St. Julien, 2005). Networks are said to be inherently more flexible than linear or hierarchical structures because their structure facilitates movement from node to node in a non-linear way, including skipping adjacent nodes if desired (Doll, 2008). Drawing on this aspect of complex systems, we envision mathematics as a network of layers of interconnected nodes (e.g., a node might represent a particular mathematical concept, or a related representation, connected to other nodes in the network) of the mathematical concept, and the learning of mathematics as making connections between the different nodes. In that sense, the richness of connections can be related to ‘learning mathematics with understanding’, and the paucity of connections to ‘finding mathematics difficult to understand’. A second aspect of complex systems related to this paper is that these systems are generative and adaptive as a result of interactions between elements in the system (St. Julien, 2005). This relates to the use of resources (by students in our case) and their (co-)design of their curriculum pathways. The use of digital resources and digital interfaces means that nodes and connections can be added (by students or teachers) to support student learning.

Hence, connectivity links with non-linear models of mathematics learning. These models recognize that students build their understanding in diverse ways. Hiebert and Carpenter (1992) proposed that it is essential to make connections in mathematics, if one intends to develop mathematical understanding and knowledge. According to them, understanding can be defined as follows:

…the way information is represented and structured. A mathematical idea or fact is understood if its mental representation is part of a network of representations. The degree of understanding is determined by the number and strength of the connections. (p. 67).

Further, it has been shown (e.g., Blömeke & Delaney, 2012) that connecting concepts, processes, and representations increase awareness of underlying mathematics structures and concepts for students and teachers. And yet, we find that the structure of print-based curriculum resources often obscures the connections that can be made. That is, curriculum documents and related (text) resources tend to use a hierarchical, linear sequence of chapters organized by strand (i.e., geometry, algebra etc.). This structure offers little support to educators in connecting mathematical ideas or in helping students make these connections.

Following this line of thinking, inspired by the concept of ‘making connections’ to support student understanding of mathematics, in an early paper Pepin and Haggarty (2011) used the concept to analyze mathematics textbook tasks in England, France and Germany with respect to connectivity. They argued as follows:

particular tasks provide different (mis)representations of mathematics for their students in school textbooks, in particular with respect to connectivity. Despite perhaps gaining proficiency at certain kinds of procedures and tasks, some students may well have gained at best a fragmented sense of the mathematics and understood few if any connections that tie together the procedures they had studied in textbooks. It can be argued that through these disconnected activities students are likely to develop perspectives on mathematics that may impede them in their use and acquisition of other mathematical knowledge. Whereas through some tasks, students are inundated with skills, procedures and disconnected mathematical knowledge, in others students are allowed to develop an appreciation of its interconnectedness and generalizable nature. (p. 13).

Moreover, it is important to note that connectivism has received extra impulse as an epistemological position due to the increase of digital technology tools and digital curriculum materials (e.g. e-textbooks). Learning and developing understanding changed from an “internal, individualistic activity” to something that can also reside outside the person, “within an organization, or database” (Siemens, 2005, p. 56), or indeed in collaboration with others. However, as Hoyles and Lagrange (2010) pointed out (quoting Artigue), connectivity does not necessarily imply collaborative work, and collaborative work does not necessarily imply better mathematics learning. They foresaw a big challenge in clarifying the relation between connectivity, collaboration and understanding.

In mathematics education, in particular in connection with computers, Internet and digital curriculum, and open educational resources, the concept of ‘connectivity’ has become a buzzword. For example, in the 17th ICMI study, Hoyles and Lagrange (2010) emphasized the influence of computers and informatics on mathematics and its teaching and learning, using ‘connectivity’ as a permeating theme. They identified different types of connectivity: (1) technological; (2) social; (3) cognitive; and (4) theoretical. Moreover, efforts in many countries emphasize making connections among disciplines, such as science and mathematics, for example. In particular, curriculum reforms in science and mathematics education refer to the need to foster students' understanding of and appreciation for the connections between these subject areas as well as their applications (e.g., Mehmetlioglu & Ozdem, 2014).

Regarding the **curriculum**, the design of learning environments and curricula poses challenges in view of emerging digital technologies: which kinds of tools should be used and aligned for the benefit of student learning? These tools not only shape the mathematical knowledge developed, but moreover the practices of teaching and the modalities of learning (Hoyles & Lagrange, 2010). Theoretical frames and tools for design are needed to guide the design of these environments and curricula.

In this respect I have been inspired by the development of learning progressions (e.g., Confrey et al., 2017), in particular those that are non-linear and that acknowledge that as learning takes place, the anticipated pathway may be altered (e.g., van den Heuvel-Panhuizen, 2008). This seems particularly relevant in higher education mathematics in innovative environments. At school level, and as an example of how this links to the learning progressions, Confrey and team (2012) combined 18 research-based learning trajectories into a curriculum map for Grades 1–8 mathematics learning. These trajectories are associated with curriculum standards, with each standard represented as a hexagon connected to other standards. They argued that this curriculum map provides guidance to educators without specifying paths to follow.

In summary, it can be argued that the notion of ‘connectivity’ can be regarded as a key principle for mathematics learning (and teaching) in innovative environments. Whether at the level of understanding mathematics learning (in general), or at the level of supporting curriculum resources (and their quality), or at the level of designing for ‘learning mathematics with understanding’ (e.g., learning progressions), making connections (at different levels) seems to be one of the main ingredients for the design of coherent learning experiences.

(2) Resources and their use

Trouche et al. (2019), in a recent book, explained the ‘resource approach to mathematics education’. Without going into further detail about the different use of the ‘resource approach’ (in terms of its connection with the Documentational Approach to Didactics), it is evident that the term ‘resource’ has been used in many studies. Broadly, we can distinguish between those studies that investigate teachers’ use of resources (e.g., Pepin et al., ), and students’ use of resources (e.g., Kock & Pepin, 2018; Pepin & Kock, 2019, 2021). In the three studies I investigate, following Pepin and Gueudet (2018), we distinguish between (a) curriculum resources (material, text, or digital); (b) social/human resources; and (c) cognitive resources of mathematics. In the context of the study on students’ use of resources, we have also added (d) general resources as an additional category: these were the resources students choose beyond the ones proposed by the university.

Many studies lean on the concept of (digital) curriculum resources to study what kinds of resources and curriculum materials teachers have used or are developing (e.g., Remillard, 2005). Fewer studies delve into student use of the available resources; amongst them is the study by Anastasakis et al. (2017), which explores how students have access to and use (for which purpose) the resources for their study of mathematics. Results showed that students mainly used those resources that helped them to pass the tests. Inglis et al. (2011) investigated the ways in which undergraduates used optional resources in a typical blended learning environment. They claimed that “the general strategy a student adopted was related to their academic achievement, both in the multivariate calculus course, and in their degree program more widely” (p. 490).

In terms of differences between digital curriculum resources and digital (educational) technologies, we proposed (Pepin et al., 2017a) a view of the main differences as being the particular attention that digital curriculum resources pay to the following:

- The aims and content of teaching and learning mathematics.
- The teacher’s role in the instructional design process (i.e., how teachers select, revise, and appropriate curriculum materials).
- Students’ interactions with digital curriculum resources in terms of how they navigate learning experiences within a digital environment.
- The impact of digital curriculum resources in terms of how the scope and sequence of mathematical topics are navigated by teachers and students.
- The educative potential of digital curriculum resources in terms of how teachers develop capacity to design pedagogic activities (p. 647).

This perspective makes sense, as we lean on *curriculum* resources (e.g., textbooks) as being those materials that are related to the (mathematics) curriculum, whether it is a one-off worksheet, or a test to assess a particular part of the curriculum (in terms of topic or grade), or a full-blown curriculum program.

It is the attention to sequencing—of grade-, or age-level learning topics, or of content associated with a particular course of study (e.g., algebra)—so as to cover (all or part of) a curriculum specification, which differentiates digital curriculum resources from other types of digital instructional tools or educational software programs. (p. 647).

Hence, in our studies curriculum resources would include text resources (e.g., textbooks, teacher guides, worksheets, tests), other material resources (e.g., manipulatives, such as base ten blocks or Cuisenaire rods, used for a particular part of the curriculum), and digital/ICT-based curriculum resources (e.g., interactive e-textbooks).

(3) Curriculum design

In this section I distinguish between (a) teachers and (b) students as (co-) designers of their curriculum, and subsequently what I mean by ‘co-design’. Moreover, as the word curriculum has already been used a number of times earlier in the paper, I would like to be more explicit about how I conceptualize it.

Drawing on van den Akker (2003), the term *curriculum* can be understood as essentially a plan for student learning. The curriculum can be viewed at different *curriculum levels* (e.g., nano, micro, meso, macro, supra levels) and at each level there are different *curriculum products*. In terms of teacher (co-)design, we work at the so-called micro level, that is, with the teacher (and the student) in the mathematics classroom, and the curriculum products are, for example, the teaching plan, instructional materials, modules, courses, textbooks.

The notion of student (co-)design has been inspired by participatory approaches to curriculum design. I concur with the view that participatory approaches to curriculum design have focused on how teachers can enact mandated curricula, which is likely to result in more relevant and robust teaching (e.g., Cochran-Smith & Lytle, 2009). In addition, researchers who study how curriculum unfolds in mathematics classrooms highlight the value of teachers working collaboratively to create an enacted curriculum in response to their needs (e.g., Boaler, 2002; Drake & Sherin, 2006; Pepin et al., 2017b). Turning to students, it has become clear that, in particular throughout the pandemic, teachers and students are co-designers of their curriculum, and even student groups are co-designing their curriculum activities in innovative learning environments (e.g., Pepin & Kock, 2021). Hence, here I acknowledge the situated work of students (and teachers).

From research on curriculum innovation (e.g., Fullan, 2007; Van den Akker & Nieveen, 2017) we know that there are different processes at stake that require coherence and consistency, integration, and alignment, in the choice and (re-) design of teaching and learning e-resources (Pepin et al., 2017a) according to what are perceived as quality criteria, as follows: the appropriation of those e-curriculum resources by teachers (Pepin et al., 2017b) and students (Pepin & Kock, 2019); the enactment of designed learning progressions (Confrey et al., 2017); the development and provision of professional development activities (based on those e-resources) for teachers (Remillard, 2005), to name but a few.

One of the key phrases in curriculum development is ‘curricular coherence’. Whilst there are various approaches to defining curricular coherence (see Confrey et al., 2017), Confrey et al. argued that the weakness in all these approaches is that “they do not explicitly acknowledge or provide for a role for the learner” (p. 718). Pointing to the concept of a “learning trajectory”, they contended that “learning trajectories describe the range of ideas that are likely to surface if the teaching and learning is student-centered and the tasks successfully elicit thinking and support its development” (p. 718). Using this concept (instead of the logical structure of the discipline) as a principle, they then defined coherence (and consistency) in a way that was different from previous definitions, which “brings new understandings of, and support for, student learning, to the forefront of curricular design” (p. 719). They defined “learner-centered curricular coherence” as follows:

an organizational means to promote a high likelihood that each learner traverses one of many possible paths to understanding target disciplinary ideas. The goal is that students achieve demonstrable and justifiable proficiency in the meanings, relationships, and utility of those target ideas by building on and continuously broadening and modifying their ideas and experiences. (p. 719).

In the curriculum development literature, frames are provided for the purpose of developing coherence amongst the different aspects of the curriculum. One of them is the ‘curricular spider web’ (van den Akker, 2003) as a useful way to visualize the connections among the various aspects of the curriculum (see Table 2), relating various curriculum components and associated core questions, such as the following: rationale (Why are students expected to learn *this*?); aims and objectives (Towards which goals are students expected to learn?); content (What are students expected to learn in this module/trajectory?); learning activities (How are students expected to learn *this*? What learning experiences would make this learning possible for students?); teacher and student role (What is the teacher’s and/or student’s role in this process?); materials and resources (Which resources do students (and teachers) need to facilitate the accomplishment of the learning goals?); grouping (With whom are students expected to learn?); location (‘where’, e.g., online, face-to-face, are students learning?); time (When are students learning?); assessment (How will student learning, in the short and long terms, be assessed?) (adapted from Thijs & van den Akker, 2009, see Pepin & Kock, 2019).

The rationale serves as a central link, connecting all other curriculum components in the web. The spider web illustrates a familiar expression: every chain is as strong as its weakest link. In other words, there has to be a balance between the components in order to provide overall coherence. At the same time a spider web is relatively flexible. Hence, in order to develop coherence and consistency (e.g., within the student learning progression), ideally, all aspects should be connected to each other in a balanced way.

We used these components (and questions) in a previous study (Pepin et al., 2017a, b), to analyze how teachers, and professional designers, might design curricula. Here I suggest that these ten questions together can lead to *flexible design principles* that should underpin each student curriculum trajectory. If the student (co-)designs his/her own learning trajectory (i.e., nano-curriculum), within the larger frame of the challenge/project, or the course provided by the teacher/university (i.e., the micro-curriculum), these are the questions s/he has to answer for his/her (co-)design.

Concerning the notion of student (co-)design, it can be argued that when students develop their own learning trajectories (in innovative learning environments), the same principles of curricular coherence (as in teacher co-design) must apply, as student (co-)design is always within the frame of the larger (micro/meso) design of learning that the teacher or university provides. For example, authorities in a university (or team of teachers within that university) can decide to have an innovative Challenge-Based education approach as the basis for their curriculum, and they then provide modules/courses and curriculum resources for the student, to be able to (co-)design their curriculum trajectory. Hence, student (co-)design is always framed by the teacher’s (co-)design of the larger learning environment, including the associated resources.

Concerning teacher curriculum (co-)design, whilst for decades research and scholarship (on factors affecting curriculum implementation) has argued for an increased involvement of teachers in shaping the learning scenarios and trajectories in their own classrooms (e.g. Ben-Peretz, 1990), in many countries teachers now actually (co-)design, customize and appropriate conventional and digitally enhanced learning materials and curriculum resources. This (co-)design work is typically conducted in collectives of classroom teachers and teacher educators (e.g., Pepin & Kock, 2019). Recent technological developments (e.g., new web-based curriculum resources; open resources) have changed the nature of teacher (co-)design work. In mathematics education, selected studies emphasize the relational aspects of (co-)design work (e.g., Pepin, et al., 2017b), or teacher curriculum design within the context of educational reform and change (e.g., Trouche et al., 2019), to name but a few.

In terms of research carried out in the field of curriculum design, there has been relatively little research in terms of students as (co-)designers of their curriculum. Whilst the curriculum is constructed with the learner as its central focus, the voice of the student is largely excluded from the curriculum design and implementation process (Bron & Veugelers, 2014). However, from existing research, student (co-)design of their own curriculum is seen as a way to engage students more and increase their enthusiasm about their own learning, and to empower them to develop their own learning trajectories. Some claim that self-regulated (and enthusiastic) students will perform better (e.g., Zimmermann & Schunk, 2001).

Pepin and Kock (2019, 2021) investigated students’ own perceptions of how they managed their learning and studying, in terms of their selection, use, sequencing and alignment of curriculum resources, and how they gave meaning to these self-created paths. These were called *Actual Student Study Paths* (ASSP), namely, students’ self-reported study paths, perceived in terms of their resource selection and organization. In that first study selected study paths for the first-year courses of Calculus and Linear Algebra were outlined (Pepin & Kock, 2019). By ‘use of resources’ we denoted the following: which resources students chose amongst the many on offer, and for what purpose (e.g. revision); the ways they align, sequence and orchestrate them (e.g. first lecture, then checking the textbook); and which ones seem central to achieve particular study goals (e.g. for weekly course work, for examinations).

In order to distinguish between the different notions (e.g., learning progression; student study path), I use the notion of ‘curriculum trajectory’ in the following way: a ‘curriculum trajectory’ encompasses the pathway through the whole study program, as well as the learning progression of particular topic areas.

In summary, curriculum design is a complex process that has been traditionally left to expert designers, and more recently also to teachers. However, it is argued that with the influence of digital resources and innovative learning environments, students have access to a more prominent role as (co-)designers of their curriculum trajectories.

## Method

In order to be able to answer the research question, I selected three studies in which I have been involved, on the basis that they corresponded to the three areas I intended to explore with respect to connectivity: (1) student design of their own study paths with digital tools (Pepin & Kock, 2019, 2021); (2) (teacher) design of classroom activities and lesson design (Pepin et al., 2017b); and (3) curriculum resources that support student learning (Gueudet et al., 2016).

For the purpose of investigating these studies (and their results) with respect to connectivity, I used my understanding of connectivity as an analytic tool for the analyses of the three studies. First, I analyzed the studies with respect to the following questions: What kinds of connections were made (e.g., by students in their design of the study paths, by the designers of digital curriculum resources) to enhance student learning? Which resources were used for making connections? Second, I made comparisons across the studies, for the purpose of answering the research question:

In which ways can *connectivity* (as a design principle) support students in (co-)designing (and enacting) their mathematics curriculum in innovative learning environments, and how can it support student interactions with resources in order to ensure curricular coherence?

## The studies and discussion of results with respect to connectivity

In this section I present the three studies that I used, for the purpose of analyzing and subsequently portraying the different connections made.

(a) Students as designers.

In this study Kock and Pepin (2018), Pepin and Kock (2019, 2021) we actually asked undergraduate mathematics students about their use of (digital) resources, in three different courses (Calculus, Linear Algebra, Bachelor End project based on the Challenge-Based Learning approach). We used a case study approach to investigate what kinds of resources were selected by students working on their two mathematics courses (Calculus, Linear Algebra) and the challenge-based projects they did as bachelor end projects. We were particularly interested in how they used and orchestrated their chosen resources. Results showed that the students working on Challenge-Based-Learning projects used more resources (e.g., Khan videos; WhatsApp with friends) outside the realm of curriculum resources (offered to them in traditional courses), and the teacher became the main ‘resource’ for monitoring and stimulating progress. Students’ Actual Student Study Paths were iterative/cyclical (see Pepin & Kock, 2021). This was in contrast to the ‘linear’ study paths found, e.g., in traditionally taught Linear Algebra courses. In the blended-learning course (Calculus) students had an abundance of resources (most of them digital) to choose from, and they felt lost in this environment. The connections that students made were the following:

- to their peers, and to the teacher;
- to the textbook/reader (provided by the university);
- to the resources provided for the course (by the course leader);
- to resources outside the university (e.g., Khan academy);
- to their family members (e.g., asking for help with tasks);
- to the stakeholders of the course (e.g., in case of the challenge-based bachelor end projects).

(b) Teacher (and student) co-design of classroom activities.

In this section I outline that whilst originally it was entirely a teacher’s task to design curriculum materials and activities beneficial for student learning, with the emergence of digital resources there is now a shifting emphasis in role division between teachers and students.

For the purpose of this paper I have chosen the study by Pepin et al. (2017b) to illustrate how connectivity plays out in (teacher) design. Results showed that digital resources in particular offered incentives and increasing opportunities for co-design by teachers and students.

In this study we drew on two different environments and scenarios:

- The first environment was the French Sésamath association, where selected French mathematics teachers worked collectively on the design of a grade 10 e-textbook (Gueudet et al., 2016). At the individual level, we also worked with teacher Vera as she engaged with digital resources in the preparation of/for her teaching, in particular with Sésamath resources.
- The second environment was the European project PRIMAS (promoting inquiry-based learning in Mathematics and Science across Europe—see Sikko et al., 2012) in Norway, where selected teachers worked collectively on the re-design and appropriation of developed resources (developed by professional designers). At the individual level, we also investigated the work of teacher Cora, as she interacted with digital (and traditional) PRIMAS resources. Drawing on these two different collective environments, and two individual teacher cases working within these environments, we investigated these mathematics teachers’ co- and re-design processes across a range of contexts and curriculum formations. The focus was on how digital resources can help to develop teacher design capacity.

One of the conclusions from the studies was that, in particular, digital resources offered new opportunities for re-design and ‘design-in-use’ (Pepin et al., 2017b). For example, in terms of ‘design-in-use’, teachers could interact differently with their students and more flexibly adapt the tasks and activities according to students’ individual in-the-moment needs. In terms of re-design and appropriation, there was a wide range of quality interactive digital resources available, often designed by specialist/professional designers.

For the present paper, I identified the connections that teachers made when designing tasks and lessons. These are also likely to be relevant issues for students as (co-)designers, when they choose particular digital curriculum resources for their curriculum trajectory and the selection of learning activities. These may be relevant to the following aspects:

- to the **curricular goal/s** for mathematics teaching/learning (as intention for the design);
- to the **products** that students are expected to produce/create in order to demonstrate achievement of these goals;
- to the **types of tasks** that offer students opportunities to create these products;
- to the **classroom activities** that may build students’ capacities to tackle these tasks;
- to particular **learning goal**s (e.g., differentiation);
- to the **coherence of mathematical thinking** and processes (**sequencing**—what do students need first before they can do that, when students work on the mathematics);
- to the **curricular ‘timing’** of mathematics topic areas in the curriculum (e.g., grades);
- to the **grouping** (e.g., with whom students should work on the mathematics);
- to the **audience**/use (e.g., who will use the resource: student/pupil, teacher, student teacher);
- to the **context** (where will the resource be used, e.g., at home, in school, on the way to work, on the Internet).

(c) Analysis and design of curriculum resources

It has been well documented that textbooks and (other) curriculum resources are crucial ingredients of every (mathematics) lesson, and they are commonly so used in mathematics classrooms across the world (Valverde et al., 2002). While being intrinsically linked to the intended curriculum, they are often seen as the most influential source for the enacted curriculum, the link between educational policy and classroom practice.

Textbooks are commonly charged precisely with the role of translating policy into pedagogy. They represent an interpretation of policy in terms of concrete actions of teaching and learning. Textbooks are the print resources most consistently used by teachers and their students in the course of their joint work. (Valverde et al., 2002, p. viii).

Because of their centrality in the teaching–learning process, policy makers have often conducted their envisaged reforms through or “by the book” (Ball & Cohen, 1996).

As textbooks turned into e-textbooks, e-textbooks may help educational innovation as they can support teachers in enacting renewed curriculum intentions in classroom processes, and they may help students to weave their way through the curriculum. At the same time, textbooks may also hinder real innovation as they limit teachers’ opportunities to (re)design the curriculum and to develop curriculum design capacity (e.g., Pepin et al., 2017b), by an overdose of detailed scripts that reduce teachers to ‘technical slaves’ (Ball & Cohen, 1996). E-textbooks have been heralded as being interactive and in supporting teachers in their everyday classroom practices, as well as in their curriculum (re-) design, through innovative and collaborative work with colleagues (Pepin et al., 2017a). They have also been announced as ‘student books’, that is, curriculum resources with which students can learn autonomously (e.g., Wood, 2016). This raises the question whether e-textbooks increase opportunities for co-design by students (and teachers). At the same time, access to useful and beneficial subject-didactical resources does not always lead to innovative practices and to learning mathematics with understanding.

In an earlier handbook chapter (Pepin et al., 2016), we defined e-textbooks (p. 644). For the purpose of this paper I have slightly modified that definition, to become the following:

E-textbooks can be defined as an evolving structured set of digital resources, dedicated to teaching and learning, initially designed by different types of authors, but open for re-design by teachers and/or students, both individually and collectively.

Moreover, we proposed to distinguish between three kinds of e-textbooks, as follows:

1. The integrative e-textbook refers to an ‘adds-on’ type model where the digital version of a (traditional) textbook is connected to other learning objects;
2. The evolving or ‘living’ e-textbook refers to an accumulative/developing type model, authored where a core community (e.g., of teachers, IT specialists) has authored a digital textbook, which is permanently developing due to the input of other practicing members/teachers;
3. The interactive e-textbook refers to a ‘toolkit’ model where the e-textbook (authored to function only as an interactive textbook) is based upon a set of learning objects, [namely], tasks and interactives (diagrams and tools) that can be linked and combined. (p. 640)

In our quest for identifying the ‘quality’ of e-textbooks and digital curriculum resources, we already recognized the notion of connectivity as an important issue. Hence, in the handbook chapter (Pepin et al., 2016) we stated that the quality of an e-textbook depends on the nature and number of connections it makes. In particular, we identified connections at two levels:

(a) *External connectivity* This type refers to the potential of linking to and between subjects/users and resources/tools outside the textbook. This may include the creation of virtual communities, connecting users with users (both teachers and students), or users and designers, or it may include links to resources outside the textbook (e.g., via web links, or platforms). More generally, we argued (ibid) that this *external connectivity* could include the following criteria:

- Connections to the national curriculum.
- Connections across grades.
- Connections with other disciplines (e.g., physics).
- Connection to the assessment system;
- Connections to other resources (files to download or websites of different kinds).
- Connections between the textbook and teacher resource systems (for synergetic effects).
- Connections between teacher and students.
- Connections in terms of teacher collective work.
- Connections between teachers using the textbook and the author/s of the textbook (Pepin et al., 2016, p. 651).

(b) *Internal connectivity* This type refers to connections made inside the e-textbook. It concerns the specific mathematical content and topic area (e.g., linking different representations of the mathematics, or associating software files or videos with the content). Internal connectivity could include the following criteria:

- Connections between different topic areas.
- Connections between different semiotic representations (e.g., text, figures, both static and dynamic).
- Connections between different software/s for carrying out a particular task.
- Connecting different mathematical concepts.
- Connecting different strategies for problem solving—which is linked to the issue of procedural vs problem-solving tasks as proposed by the textbook.
- Connecting different moments of appropriating a given concept (e.g., spiral progression, progressively deepening a concept instead of proposing a complete presentation of it in the same chapter) (Pepin et al., 2016, pp. 651–652).

In the subsequent study Gueudet et al., (2018) we went a step further. As this paper was concerned with the development of e-textbooks, we claimed that analysis (and design) of e-textbooks requires the development of a specific frame. We developed such a frame (addressing external and internal connections) based on the notion of connectivity, sampling commonly used and contrasting textbooks (according to our earlier definitions of e-textbooks), choosing a particular topic area (grade 10: functions, sampled on issues of didactical interest, e.g., different representations), and analyzing two French e-textbooks to illustrate the framework.

The results of the analyses showed that there were major differences between the two e-textbooks, in terms of potential for effecting connections:

- External connections: In one textbook (an “integrative e-textbook”—see Pepin et al., 2016), no changes to the initial e-textbook were possible for teachers or the students. However, there were possibilities for creating a personal space within the e-textbook (although it was not possible to import external resources). In the other textbook (an “evolving e-textbook”), teachers could download all the parts of the e-textbook, modify them, and integrate them into their resource systems. Teachers could also create their own sessions on a particular platform space and share them with colleagues, and they could provide individualized tasks to students. Students had the possibility of communicating with the teacher.
- Internal connections: In both textbooks (for the teaching and learning of ‘functions’) a rich network of connections was offered: connections were made between concepts, between different representations; connections between the mathematical topic studied and other topic areas, or with other disciplines. We concluded that these connections were likely to support rich mathematical activity for students with the e-textbook and to foster their development of cognitive connections. (Gueudet et al., 2018)

## Conclusions

In this section I answer the research question and I provide conclusions, both in theoretical as well as practical terms.

In this conceptual paper I have chosen to introduce and use the concept of *connectivity* as a critical feature (and principle) of curriculum design, which can be used—beyond the case of e-textbooks analysis—for supporting mathematics students (and teachers) in designing their own curriculum and increasing coherence among the different components of the (mathematics) curriculum in time of digitalization. I explored the concept of connectivity as a (design) principle for (1) making and displaying the many connections among mathematical ideas within mathematics curricula; (2) supporting university mathematics students in choosing and orchestrating the resources needed for developing their own curriculum trajectories, in other words, in (co-)designing their own curriculum.

Using previous studies for analyses and illustration purposed, I argue that it would be beneficial for students (and teachers) to effect connections at different levels:

- at a social level—across people (e.g. peer-peer, student-tutor, student-stakeholder).
- at a material level—across digital or analogue curriculum resources provided by course leaders (e.g., textbooks, course reader, video lecture), and across resources students find themselves (e.g., Khan academy).
- at a curriculum (design) level—among the curricular goals and the student goal/s, among the learning goals and the outcomes/products of their learning, and of course among the student outcomes/products and the audience, the context (in which they study), and the types of tasks and activities in which they need to engage in order to get to the desired outcomes and products.
- at a didactical level (e.g., in e-textbooks, for the purpose of the development of an enhanced mathematical understanding and coherence of their mathematical thinking)—across (physical) contexts/learning environments; across people/learning communities; across curriculum levels and representations; across students’ mathematical thinking and practices (e.g., Aguirre, et al., 2012); across mathematical representations (e.g., Gueudet, et al., 2018); across contexts (e.g., Voigt et al., 2020); and across mathematical concepts.

What I suggest is that these kinds of connections can work as a frame for students for their (co-) design of their own curriculum trajectories. The notion of connectivity, together with the connections proposed above (in particular at the curriculum design level), allow me to offer a ‘tool’ for (1) raising awareness of potential connections that can be made for enhancing the coherence of student learning and study experiences; and (2) developing curriculum trajectories for studying and learning in innovative environments where digital resources are meaningfully used. Leaning on complexity thinking, connections can be systematically considered, which is likely to help students to enhance the coherence in their (co-)designs.

Moreover, the principle of connectivity can act as support for students, in order for them to be able to develop coherent curriculum trajectories by aligning suitable curriculum resources, so that they can realize their mathematical goals in their studies. However, students need support in making connections, for example, across the different components of the curriculum (for internal curriculum coherence) (Schmidt et al., 2005); or across different mathematical representations (e.g., mediated by different digital resources), in order to develop connected and coherent mathematical understanding.

The implications of this are that beside appropriate (e-)resources, students (and teachers) need support in professional (for teachers) or peer (for students) collectives, to develop an awareness of the different aspects of connectivity and the capacity to increase internal and external curriculum coherence for enhanced understanding of the mathematics (Confrey et al., 2017). E-textbooks and digital curriculum resources offer increasing opportunities for making connections easier (e.g., because students/teachers do not have to sit in the same place to connect), and richer (e.g., because teachers and students can link to external/open curriculum resources), but only if they are aware of the beneficial opportunities for a more thoughtful design and use of the mathematics curriculum. That means that focusing on connectivity is not only useful for analysing e-textbooks and curriculum resources, and it is not only helpful for teachers, but also for students, as it could provide a window into issues of interactivity, both practically and cognitively. Indeed, raising awareness of *connectivity* could enrich the potential for student (co-)design of their own curriculum.

The argument of this paper is that the existence and availability of suitable digital curriculum resources (and technologies) imply a revision of the pedagogical landscape in terms of the ways in which students can be empowered to design their own curriculum. Especially, the notion of a *connected curriculum trajectory* should be shaped and structured by suitably connected and mathematically-didactically appropriate curriculum resources (e.g., interactive e-textbooks and engaging mathematical tasks) through digital means (e.g., platforms). Teachers may develop the intended or hypothetical learning trajectory, whereas students may design and enact the actual learning trajectory. I contend that a student designed *connected curriculum trajectory* is likely to become the focus of future research activities in innovative learning environments: this focus would connect aspects of curriculum, mathematical content, learning strategies of students, and the use of new technologies.
